# Multiobjective Optimization of a Plate Heat Exchanger in a Waste Heat Recovery Organic Rankine Cycle System for Natural Gas Engines

**DOI:** 10.3390/e21070655

**Published:** 2019-07-03

**Authors:** Guillermo Valencia, José Núñez, Jorge Duarte

**Affiliations:** 1 Programa de Ingeniería Mecánica, Grupo de Investigación en Gestión Eficiente de la Energía KAI, Universidad del Atlántico, Carrera 30 Número 8-49, Puerto Colombia, Área Metropolitana de Barranquilla 080007, Colombia; 2 Departamento de Energia, Grupo de Investigación en Optimización Energética GIOPEN, Universidad de la Costa CUC, Cl. 58 # 55-66, Barranquilla, Atlántico 080002, Colombia

**Keywords:** acquisition cost, entropy generation number, heat exchanger, multiobjective optimization, ORC, waste heat recovery

## Abstract

A multiobjective optimization of an organic Rankine cycle (ORC) evaporator, operating with toluene as the working fluid, is presented in this paper for waste heat recovery (WHR) from the exhaust gases of a 2 MW Jenbacher JMS 612 GS-N.L. gas internal combustion engine. Indirect evaporation between the exhaust gas and the organic fluid in the parallel plate heat exchanger (ITC2) implied irreversible heat transfer and high investment costs, which were considered as objective functions to be minimized. Energy and exergy balances were applied to the system components, in addition to the phenomenological equations in the ITC2, to calculate global energy indicators, such as the thermal efficiency of the configuration, the heat recovery efficiency, the overall energy conversion efficiency, the absolute increase of engine thermal efficiency, and the reduction of the break-specific fuel consumption of the system, of the system integrated with the gas engine. The results allowed calculation of the plate spacing, plate height, plate width, and chevron angle that minimized the investment cost and entropy generation of the equipment, reaching 22.04 m^2^ in the heat transfer area, 693.87 kW in the energy transfer by heat recovery from the exhaust gas, and 41.6% in the overall thermal efficiency of the ORC as a bottoming cycle for the engine. This type of result contributes to the inclusion of this technology in the industrial sector as a consequence of the improvement in thermal efficiency and economic viability.

## 1. Introduction

Natural gas is one of the most profitable fuels to replace conventional fuels, such as diesel and gasoline, around the world [1]. One of the reasons is a large number of reserves of fossil fuels around the world that are becoming considered as a suitable alternative in the industrial energy sector [2]. All devices such as heat exchangers implicate energy losses during the process, which is equivalent to increasing the total operational costs and reducing the energy performance of the system. Therefore, improvement in the thermal performance of a gas engine was proposed by incorporating an exhaust gas heat recovery system using an organic Rankine cycle (ORC) [3]. However, this proposed configuration requires a thermal oil circuit, in which the residual exhaust gas is used to evaporate the organic fluid with the help of a thermal oil that regulates the amount of heat transferred and decreases the ORC evaporation temperature.

The optimization of equipment used for waste heat recovery (WHR) has been studied by many researchers using different methods and formulations. Technical challenges, such as the high acquisition costs and entropy generation inside the heat exchanger, represent an improvement opportunity to increase ORC performance [4]. In these cases, mathematical tools can be used to find the best solutions through a stochastic search according to the objective selected. Holland [5] and De Jong [6] introduced the concept of genetic algorithms in publications, although these were not applied to the heat transfer field of knowledge.

Several researchers have applied optimization techniques to design industrial equipment using thermodynamic and economic approaches, specifically in heat exchangers in the last years. Martin et al. [7] used a dimensionless function proportional to the sum of annual investment costs and operating costs, where the minimum of this function made it possible to determine the optimal Reynolds number, which depends on the type of heat exchanger chosen. In other research, Niclout et al. [8] describe an optimization problem in which objective functions, such as manufacturing cost and heat exchanger volume, as well as operating and manufacturing constraints were studied considering as decision variables the geometric parameters of the fins. To solve this problem, the author developed nonlinear programming of mixed integer numbers, like other options of the solution. Nevertheless, his work is limited by not considering the thermodynamic parameters involved in the system but only the geometric parameters, although this means a possible over-dimensioning of the equipment. 

An economic point of view has been studied in heat exchanger design. Selbaş et al. [9] used genetic algorithms to design a shell and tube heat exchanger that varied design variables, such as the outer tube diameter, the tube arrangement, and the outer shell diameter, among other geometric parameters to obtain the optimal heat transfer area for the desired configuration using the logarithmic media temperature difference (LMTD) method. Likewise, using genetic algorithms, Ozkol et al. [10] determined the optimal geometry to obtain the minimum area and acquisition cost, considering the limits of performance and operation, in a specific thermal application. These formulations use similar principles to those used in this research, but they do not consider economic indicators and, moreover, operational limitations since they differ completely from this study. 

Muralikrishna et al. [11] proposed a methodology to determine the feasible range of a heat exchanger design based on pressure drop criteria. Although, this research does not give concrete results about the design and sizing of this equipment because it only developed a valid design range to determine important knowledge about the limitations for designing this equipment widely used in the industry.

These results indicate a global concern to improve the thermal performance of this equipment and, thus, the global efficiency of the systems. Jarzebski et al. [12] calculated the minimum cost of the operation and maintenance of a plate heat exchanger based on constant geometry and variable flow of the working fluid, and a simple analytical correlation for the pressure drop is presented. In this case, the authors did not apply a genetic algorithm. On the other hand, Zhu et al. [13] performed geometric optimization of a plate heat exchanger in a geothermal application through the use of iterative programming to determine the optimal configuration that satisfied the objective (minimum area). This study has concrete similarity to the current study because the decision variables are similar. However, the author did not perform multiobjective optimization, which restricts the analysis of their investigation. Ahmadi et al. [14,15] performed a multidimensional thermo-economic analysis using multiobjective optimization of an integrated biomass system using a genetic algorithm. The integrated system is an ORC system cooled through an absorption chiller, a hydrogen production unit, a domestic water heater, and a reverse osmosis desalination system. For this purpose, the authors used the design variables as selection criteria, while the total costs and energy efficiency indicators of the system were selected as objective functions. Additionally, a sensitivity analysis was performed to evaluate the effect of the decision variables on exergy destruction rate, CO_2_ emissions, and energy efficiency, which is similar to the approach presented in this paper but with different objective functions.

Wang et al. [16] performed multiobjective optimization of the ORC condenser using a genetic algorithm, where the pressure drop and the heat transfer area were minimized under constant heat transfer conditions, resulting in a series of optimal solutions presented in a Pareto frontier. However, the author did not base their optimization on a thermo-economic analysis, which indicates that the effect of equipment costs on the results obtained was not considered. Consequently, minimization of equipment costs and the entropy generation number, considering geometrical parameters of the parallel plate heat exchanger (ITC2) without its effect on the heat transfer capacity of the equipment and reducing system performance, had not been developed considering some energy indicators, such as global energy conversion efficiency and energy efficiency, which decrease due to the irreversibility of the process and limits its application and commercial feasibility [17].

From these results and their limitations, the main contribution of this research is the multiobjective optimization of a plate heat exchanger, which was used to evaporate the organic fluid in an ORC as a WHR system from a natural gas engine using the NSGA-II genetic algorithm, and a detailed thermodynamic model of the heat exchanger. The most sensitive variables of the system are determined, and the optimal configuration is selected to obtain the minimum acquisition cost and entropy generation number. In addition, the influence of the design parameters of the thermodynamic cycle, such as the evaporator and condenser pinch temperatures, the turbine and pump efficiency, and different working fluids were studied.

## 2. Description and Model of the System

### 2.1. Description of The Waste Heat Recovery System

**Figure 1:**
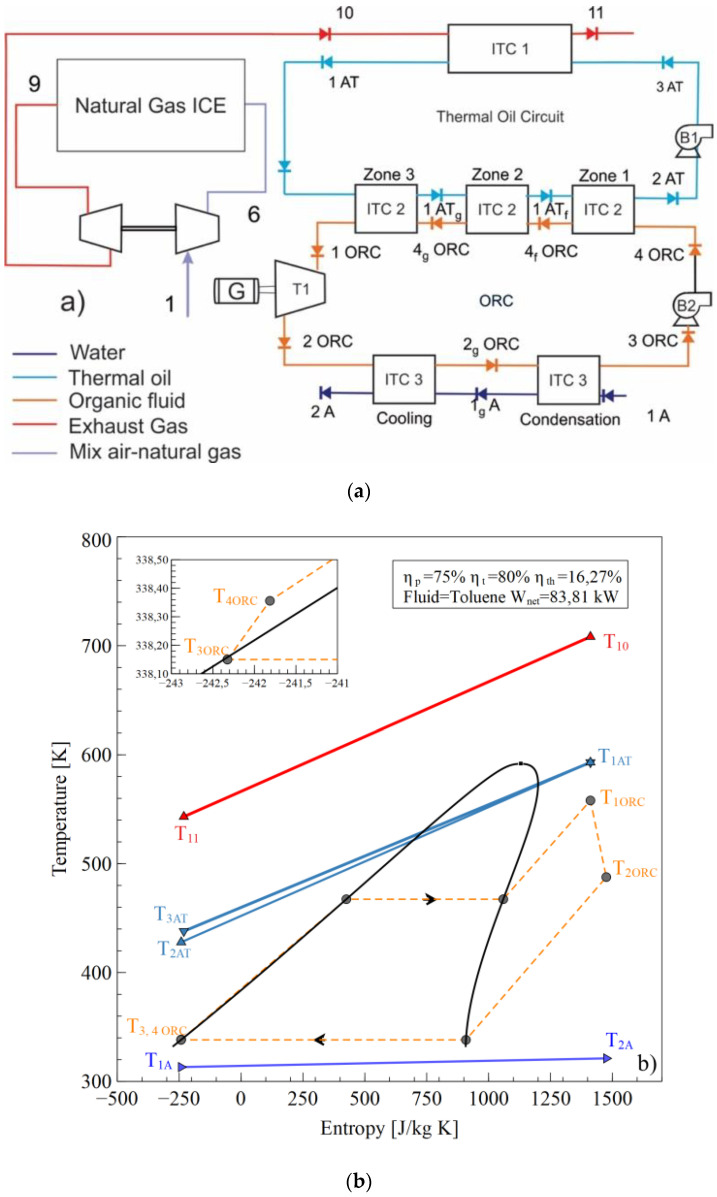
Waste Heat Recovery ORC system, (**a**) Physical structure, (**b**) T-s diagram.

The configuration shown in Figure 1a [18] operates as the T–s diagram in Figure 1b is presented. An air-fuel mixture (1) is delivered to the internal combustion engine (ICE), which is compressed in the compressor stage of the turbocharger (6) to achieve the conditions required for combustion in the cylinders. The gases at the outlet of the exhaust manifold (9) expand in the expansion stage of the turbocharger to reach the stream (10) and are disposed the environment (11) after transferring the energy in the heat exchanger tube and shell (ITC1) with the thermal oil (3 AT), which flows through the thermal oil circuit pumped by Pump 1 (B1). 

The thermal oil (1AT) transfers the energy to evaporate the ORC working fluid in the (ITC2) to obtain the superheated steam in (1ORC) to generate thermal power in the turbine (T1) and electric energy in the generator (G). In the evaporator (ITC2), three zones are presented: the preheating zone (zone 1), the evaporation zone (zone 2), and the superheating zone (zone 3). The working fluid expands until the low pressure (2ORC) is inputted in the condenser heat exchanger (ITC3), where the fluid is fully condensed to the saturated liquid phase (3ORC). Condensation is conducted in two successive stages called condensation (1A to 1gA) and cooling (1gA to 2A). Subsequently, the working fluid pump (B2) increases the fluid pressure to the evaporating pressure of the ITC2, which finishes the WHR system from the natural gas engine by ORC using toluene as the working fluid [19,20,21,22]. 

### 2.2. Energy Analysis

The components of the WHR system were studied under the assumption of an open system operating under steady state conditions. Consequently, the mass and energy balance must be calculated, as shown in Equation (1) and Equation (2), respectively.

(1) $$\sum {\overset{\cdot }{m}}_{\mathrm{in}}-\sum {\overset{\cdot }{m}}_{\mathrm{out}}=0$$

(2) $$\sum {\overset{\cdot }{m}}_{\mathrm{in}}h_{\mathrm{in}}-\sum {\overset{\cdot }{m}}_{\mathrm{out}}h_{\mathrm{out}}-\sum \overset{\cdot }{Q}+\sum \overset{\cdot }{W}=0$$

Some performance indicators were calculated to assess the WHR systems, such as the thermal efficiency of the configuration $(\eta_{I,\;c}$), heat recovery efficiency $(\varepsilon_{\mathrm{hr}}$), and overall energy conversion efficiency $(\eta_{I,\;\mathrm{global}}$) [23]. The thermal efficiency of the configuration (Equation (3)) is the ratio of the net power output of the ORC (${\overset{\cdot }{W}}_{\mathrm{net}}$) recovered, with respect to the waste heat. In this case, the net power output of the ORC is the turbine power output (T1) less the energy consumption of the thermal oil pump (B1) and organic fluid pump (B2).

(3) $$\eta_{I,\;C}=\frac{{\overset{\cdot }{W}}_{\mathrm{net}}}{{\overset{\cdot }{Q}}_{G}}$$

In addition, the waste heat recovery efficiency (Equation (4)) is the heat recovered from the exhaust gas engine line, with respect to the maximum heat available to be removed, and the overall energy conversion efficiency (Equation (5)) is the relation between the net power output and the available waste heat.

(4) $$\varepsilon_{\mathrm{hr}}=\frac{{\overset{\cdot }{Q}}_{G}}{{\overset{\cdot }{m}}_{10}\cdot C_{P10}\cdot \left(T_{10}-T_{0}\right)}$$

(5) $$\eta_{I,\;\mathrm{overall}}=\eta_{I,\;C}\cdot \varepsilon_{\mathrm{hr}}$$

Also, to consider global indicators involving engine operation conditions, the absolute increase of engine thermal efficiency (Equation (6)) was calculated as a measurement of the net power output to the energy supply by the natural gas in the engine.

(6) $$\Delta \eta_{\mathrm{thermal}}=\frac{{\overset{\cdot }{W}}_{\mathrm{net}}}{{\overset{\cdot }{m}}_{\mathrm{fuel}}\cdot \mathrm{LHV}}$$

Because additional power is delivered in the heat recovery system over the engine power, there is a break-specific fuel consumption (BSFC), which is determined by Equation (7), and generating more power with the same fuel consumption decreases the specific fuel consumption of the engine, which is calculated as follows in Equation (8) [24].

(7) $${\mathrm{BSFC}}_{\mathrm{ORC}-\mathrm{engine}}=\frac{{\overset{\cdot }{m}}_{\mathrm{fuel}}}{{\overset{\cdot }{W}}_{\mathrm{engine}}+{\overset{\cdot }{W}}_{\mathrm{net}}}$$

(8) $$\Delta \mathrm{BSFC}=\frac{\left|{\mathrm{BSFC}}_{\mathrm{ORC}-\mathrm{engine}}-{\mathrm{BSFC}}_{\mathrm{engine}}\right|}{{\mathrm{BSFC}}_{\mathrm{engine}}}\cdot 100$$

### 2.3. Exergy Analysis

The specific exergy for the process states is calculated by neglecting the variations of kinetic and potential energy, resulting as shown in Equation (9).

(9) $$\mathrm{ex}=\left(h-h_{0}\right)-T_{0}\left(s-s_{0}\right)$$

The chemical exergy of the exhaust gases as a product of combustion (stream 10), is defined by Equation (10). One has a mixture of gas products of the combustion of natural gas. The chemical exergy of the gas mixture is given by Equation (10).

(10) $${\mathrm{ex}}_{G}^{\mathrm{ch}}=\sum_{i=1}^{n}X_{i}\cdot {\mathrm{ex}}^{{\mathrm{ch}}_{i}}+R\cdot T_{0}\cdot \sum_{i=1}^{n}X_{i}\cdot {\mathrm{lnX}}_{i}$$

Also, the exergetic efficiency based on the second law of thermodynamics ($\eta_{\mathrm{II},\;\mathrm{ORC}}$) is calculated with Equation (11), additionally expressed as a function of the destroyed exergy (${\overset{\cdot }{E}x}_{\mathrm{Destr}}$) using Equation (12).

(11) $$\eta_{\mathrm{II},\;\mathrm{ORC}}=\frac{{\overset{\cdot }{E}x}_{\mathrm{Produced}}}{{\overset{\cdot }{E}x}_{\mathrm{supplied}}}$$

(12) $$\eta_{\mathrm{II},\;\mathrm{ORC}}=1-\frac{{\overset{\cdot }{E}x}_{\mathrm{Destr}}}{{\overset{\cdot }{E}x}_{\mathrm{supplied}}}$$

### 2.4. Modeling of The Plate Heat Exchanger

The parallel plate heat exchangers are devices designed to provide a large surface area of heat transfer per unit volume [25] in addition to achieving high heat transfer rates between two fluids in a small volume [26,27]. The design of this heat exchanger takes into account the phase change in the organic fluid at low temperatures [28] to select the optimal configurations that guarantee the functional operation of the equipment within the thermal oil circuit. 

**Figure 2:**
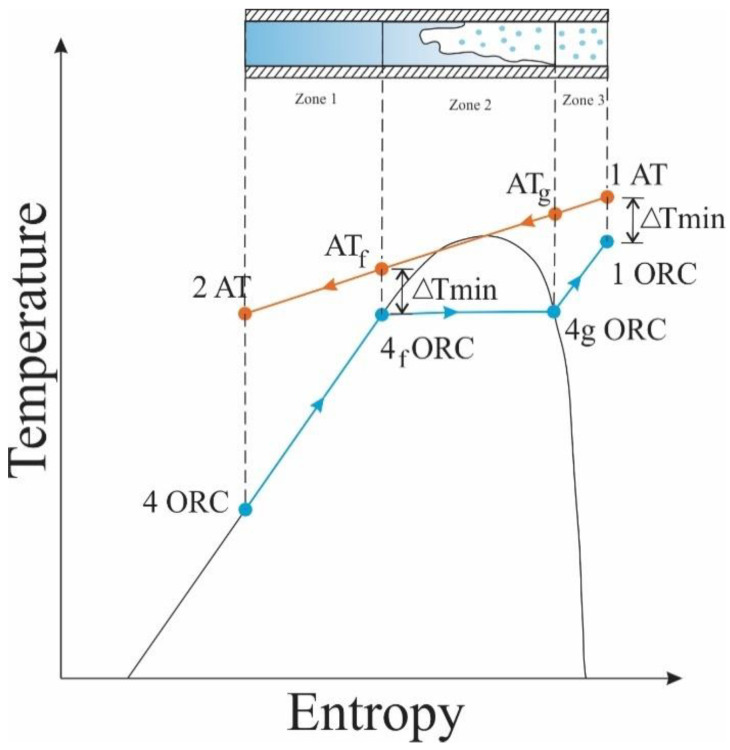
T-s diagram of the heat transfer process between thermal oil and organic fluid.

For the heat exchanger studied, Therminol 75 was used as thermal oil and hot fluid for the ITC2 design. This oil recovers a fraction of the heat available in the exhaust gas line of the MCI and transfers the energy to heat the organic fluid, which presents a phase change during the process. For this reason, the thermal design considers different stages of the organic fluid, represented by zones, to facilitate the description of the process and the design of the device, as shown in the T–s diagram in Figure 2 of the heat transfer process between the two working fluids in the ITC2. 

**Figure 3:**
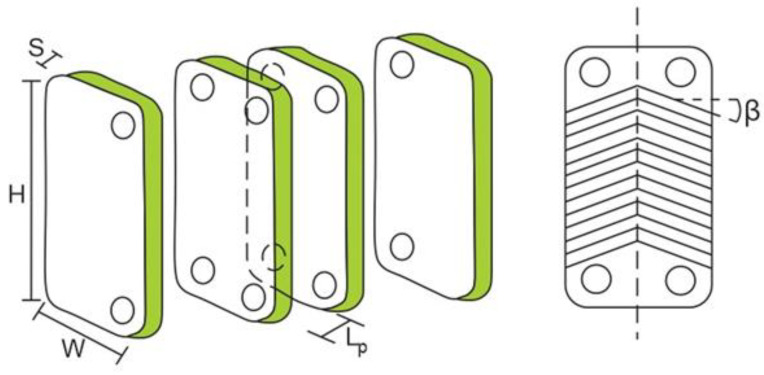
Geometric parameters of the plate heat exchanger ITC2.

The increase of plate spacing has a direct effect on the channel’s cross-section area, channel velocity, equivalent diameter, thermal oil, and the organic fluid Reynolds number in the ITC2. Generally, these parameters affect the total heat transfer area, total pressure drop, and exchanger performance [29]. In this section, the determination of geometric parameters is presented, as shown in Figure 3, which affects the number of plates and the heat transfer area of the equipment in each zone. The equations are analyzed under three zones: preheating (zone 1), evaporation (zone 2), and overheating (zone 3). 

Figure 2 shows the temperature limits in the mixing phase, where the temperature of the thermal oil at the beginning of the phase is calculated using Equation (13) with the pinch (known as the minimum temperature difference), and the temperature at which the organic fluid leaves ITC2 is calculated with Equation (14) [30].

(13) $$T_{\mathrm{ATf}}=T_{4\mathrm{fORC}}+\mathrm{pinch}$$

(14) $$T_{1\mathrm{ORC}}=T_{1\mathrm{AT}}-\mathrm{pinch}$$

The heat transfer flow is calculated for each particular zone. Equation (15) allows calculation of heat flow in zone 1, and the heat of each zone is a function of the different enthalpies presented in the process.

(15) $${\overset{\cdot }{Q}}_{Z1}={\overset{\cdot }{m}}_{\mathrm{AT}}\cdot \left(h_{\mathrm{ATf}}-h_{2\mathrm{AT}}\right)$$

The enthalpy of the organic fluid at the ITC2 inlet is calculated as Equation (16).

(16) $$h_{4ORC}=h_{\mathrm{ORCf}}-\left(\frac{{\overset{\cdot }{Q}}_{Z1}}{{\overset{\cdot }{m}}_{\mathrm{ORC}}}\right)$$

where $h_{4\mathrm{ORC}}$ and $h_{\mathrm{ORCf}}$ are in kJ/kg. The logarithmic mean temperature difference is an essential factor in the heat exchanger design, which is calculated for zone 1 as Equation (17).

(17) $$LMTD_{Z1}=\frac{\left(T_{ATf}-T_{ORC}\right)-\left(T_{2AT}-T_{1ORC}\right)}{\log\|\frac{T_{ATf}-T_{ORC}}{T_{2AT}-T_{1ORC}}\|}$$

where ${\mathrm{LMTD}}_{Z1}$ is given in Kelvin. Other relevant factors in designing the heat exchanger are the plate area and the heat transfer area in m^2^, which are calculated using Equations (18) and (19), respectively.

(18) $$A_{plate}=1\times 10^{-6}\cdot W\cdot H$$

(19) $$A_{Z1}=\frac{{\overset{\cdot }{Q}}_{Z1}}{U_{Z1\_A}\cdot LMTD_{Z1}}$$

where plate width (*W*) and plate height (*H*) are in mm, and $U_{Z1\_A}$ represents the overall heat transfer coefficient for zone 1 in Wm2K.

Through the application of Equations (20)–(31) it is possible to calculate the most relevant geometric characteristics of the equipment, such as the plate thickness, number of plates per zone, number of passes, the velocities, pressure drops in the system, and the exergy destroyed in each zone during the process

(20) $$N_{P\_Z1}=\frac{A_{Z1}}{A_{\mathrm{plate}}}+1$$

(21) $$N_{\mathrm{Plate}}=N_{P\_Z1}+N_{P\_Z2}+N_{P\_Z3}$$

(22) $$N_{\mathrm{ch}\_\mathrm{pass}}=\frac{N_{\mathrm{Plate}}-1}{2}+1$$

The velocities determine the fluid flow regimen and their characteristics. Therefore, the design considers the calculation for each zone of the dimensionless numbers of Reynolds (Equation (23)), Prandtl (Equation (24)), and Nusselt (Equation (25)) [31]. This allows us to obtain valuable information about the fluid properties, the flow characteristics, and the fluid heat transfer capacity.

(23) ReZ1_AT=vZ1_AT·DhυZ1_AT

(24) $${\mathrm{Pr}}_{Z1\_\mathrm{AT}}=\frac{\upsilon_{Z1\_\mathrm{AT}}\cdot {\mathrm{Cp}}_{Z1\_\mathrm{AT}}\cdot \rho_{Z1\_\mathrm{AT}}}{k_{Z1\_\mathrm{AT}}}$$

(25) NuZ1_AT=0,78·ReZ1_AT0.5·(PrZ1_AT)13

where the hydraulic diameter (Dh) is in m, the fluid velocity ($v_{Z1\_\mathrm{AT}})$ is in m/s, the kinematic viscosity ($\upsilon_{Z1\_\mathrm{AT}}$) in m²/s, density ($\rho_{Z1\_\mathrm{AT}})$ is in kg/m^3^, the thermal conductivity ($k_{Z1\_\mathrm{AT}}$) is in W/m·K, and the specific heat at constant pressure $\left({\mathrm{Cp}}_{Z1_{\mathrm{AT}}}\right)$ is in J/kg·K. To calculate the drop pressure at the inlet, and because of the flow within zone 1 for the thermal oil side, Equations (27) to (28) are used, which requires the velocity calculation at the ITC2 input of the thermal oil using Equation (26), where ${\mathrm{vin}}_{Z1\_\mathrm{AT}}$ is in m/s.

(26) $${\mathrm{vin}}_{Z1\_\mathrm{AT}}=\frac{{\overset{\cdot }{m}}_{\mathrm{AT}}}{\frac{\pi }{4}{\left(\mathrm{Dh}\right)}^{2}\cdot \rho_{Z1\_\mathrm{AT}}}$$

(27) $$\Delta {\mathrm{Pin}}_{Z1\_\mathrm{AT}}=\frac{1.3*\rho_{Z1\_\mathrm{AT}}}{2}\cdot \left[\frac{{\left({\mathrm{vin}}_{Z1\_\mathrm{AT}}\right)}^{2}}{100}\right]$$

(28) $$\Delta {\mathrm{PF}}_{Z1\_\mathrm{AT}}=\frac{8\cdot {\mathrm{jf}}_{Z1\_\mathrm{AT}}\cdot \rho_{Z1\_\mathrm{AT}}\cdot \left(\frac{\mathrm{Lp}}{\mathrm{Dh}}\right)}{2}\cdot \left[\frac{{\left({\mathrm{vin}}_{Z1\_\mathrm{AT}}\right)}^{2}}{100}\right]$$

where the thermal oil mass flow (${\overset{\cdot }{m}}_{\mathrm{AT}}$) is in kg/s, hydraulic diameter (Dh) is in m, and the drop pressure (ΔP) is obtained in mbar.

The total pressure drop in the heat exchanger is the total pressure loss on the thermal oil side (Equation (29)) and the total pressure drop on the organic fluid side (Equation (30)), involving the pressure drops due to flow (ΔPF) and the pressure drops at the fluid inlet (ΔPin) in each of the zones.

(29) $$\Delta P_{\mathrm{tot}\_\mathrm{AT}}=\Delta {\mathrm{PF}}_{Z1\_\mathrm{AT}}+\Delta {\mathrm{PF}}_{Z2\_\mathrm{AT}}+\Delta {\mathrm{PF}}_{Z3\_\mathrm{AT}}+\Delta {\mathrm{Pin}}_{Z1\_\mathrm{AT}}+\Delta {\mathrm{Pin}}_{Z2\_\mathrm{AT}}+\Delta {\mathrm{Pin}}_{Z3\_\mathrm{AT}};$$

(30) $$\Delta P_{\mathrm{tot}\_\mathrm{ORC}}=\Delta {\mathrm{PF}}_{Z1\_\mathrm{ORC}}+\Delta {\mathrm{PF}}_{Z2\_\mathrm{ORC}}+\Delta {\mathrm{PF}}_{Z3\_\mathrm{ORC}}+\Delta {\mathrm{Pin}}_{Z1\_\mathrm{ORC}}+\Delta {\mathrm{Pin}}_{Z2\_\mathrm{ORC}}+\Delta {\mathrm{Pin}}_{Z3\_\mathrm{ORC}}.$$

Consumption of the potential useful work in zone 1 of ITC2 is calculated by Equation (31), which represents the exergy destroyed by the system in this zone expressed in watts. Similarly, the destroyed exergy is calculated for the other ITC2 zones, facilitating the computation of one of the objective functions considered in the optimization of this equipment.

(31) $${\overset{\cdot }{e}}_{Z1}=\frac{{\overset{\cdot }{m}}_{\mathrm{ORC}}\left(\Delta {\mathrm{PF}}_{Z1\_\mathrm{ORC}}+\Delta {\mathrm{Pin}}_{Z1\_\mathrm{ORC}}\right)}{\rho_{Z1\_\mathrm{ORC}}}+\frac{{\overset{\cdot }{m}}_{\mathrm{AT}}\left(\Delta {\mathrm{PF}}_{Z1\_\mathrm{AT}}+\Delta {\mathrm{Pin}}_{Z1\_\mathrm{AT}}\right)}{\rho_{Z1\_\mathrm{AT}}}$$

## 3. Multi-Objective Optimization

### 3.1. Methodology of Design

The objective of the design of the parallel plate heat exchanger is to provide the lowest amount of entropy generated during the process [32] with the smallest heat transfer area and, therefore, the best cycle efficiencies [25]. For this reason, it is necessary to determine a geometric configuration that accomplishes the thermodynamic requirements with the lowest possible acquisition cost [33]. It is for this reason that the methodology implemented for this design is multiobjective optimization applied in various disciplines to minimize or maximize two or more functions simultaneously [34], which can be expressed as shown in Equation (32),

(32) $$\min F\left(X\right)={\left[f_{1}\left(X\right),f_{2}\left(X\right),f_{3}\left(X\right)\ldots ,f_{n}\left(X\right)\right]}^{T}$$

and is subject to restrictions $g_{i}\left(X\right)\leq 0;\;i=1,\ldots ,m$ and $h_{i}\left(X\right)=0;\;\mathrm{and}\;j=1,\ldots ,n$ within a range of criteria for $X_{k,\;\min}\leq X_{k}\leq X_{k,\;\max}$. For these systems, the calculation of the entropy generation number (EGN) is proposed using Equation (33), and the heat exchanger acquisition cost is expressed as in Equation (34) [35].

(33) $$\mathrm{EGN}=\frac{\overset{\cdot }{S}\mathrm{gen}\cdot T_{0}}{\overset{\cdot }{Q}}$$

(34) $$\mathrm{Cost}\left(\$\mathrm{USD}\right)=10000+324\cdot A_{z}{}^{0.91}$$

The results obtained by this technique display a series of geometric combinations that provide the functionality of the proposed objectives, which form a set of points called a Pareto front. In determining the most optimal points, however, a multicriteria decision technique is required to facilitate decision-making and, therefore, the optimization of the parallel plate heat exchanger.

### 3.2. Multi-Criteria Decision

The multicriteria decision method called the technique of order preference for similarity to ideal solution (TOPSIS) [36,37,38], is applied to select a point for the ideal geometrical configuration, which is a suitable method for this type of applications and is used to classify the alternatives to the Pareto solutions obtained.

This technique mathematically identifies the point with the nearest distance to the positive ideal solution with the longest distance to the negative ideal solution, conditions which are calculated by Equation (35)

(35) $$\begin{array}{c} d_{\mathrm{ix}}=\sqrt{\sum_{j=1}^{n}{\left(t_{\mathrm{ij}}-t_{\mathrm{xj}}\right)}^{2}},\;i=1,2,\ldots ,m\\ d_{\mathrm{iy}}=\sqrt{\sum_{j=1}^{n}{\left(t_{\mathrm{ij}}-t_{\mathrm{yj}}\right)}^{2}},\;i=1,2,\ldots ,m \end{array}.$$

where $d_{\mathrm{ix}}$ and $d_{\mathrm{iy}}$ are the distances from the points to the ideal positive and negative solution, respectively; $t_{\mathrm{ij}}$ is the reference value of alternative i for objective j; and $t_{\mathrm{xj}}$ and $t_{\mathrm{yj}}$ are the ideal and nonideal values, respectively. The relative proximity to the ideal solution ($S_{\mathrm{iy}}$) is calculated using Equation (36)

(36) Siy=diy(diy+dix)   0≤Siy≤1,

where the best solution is the one whose $S_{\mathrm{iy}}$ is the closest to 1.

## 4. Results and Discussion

### 4.1. Working Fluid Selection

This research mainly focused on improving the energetic and exergetic performance of the WHR system, and selection of the working fluid was oriented to the increase net power [39]. For this case, 14 preselected working fluids were evaluated in simulation to study the performance of the WHR system for the Jenbacher JMS 612 GS-N.L engine, using the simple ORC configuration under typical engine operating conditions. The results of the performance indicators studied are shown in descending order (Table 1).

The results show the MD4M and D6 fluids had the lowest net power with values of 30.84 kW and 32.04 kW. These results were near to those obtained in the ASPEN HYSYS^®^ simulation with the CATERPILLAR C32 ATAAC diesel engine [40], where a simple ORC was used to make use of the residual heat available in the engine exhaust gases.

Based on these results, acetone, cyclohexane, and toluene were the organic fluids that presented the best values in the analysis performed. Therefore, they were selected to analyze the influence they had on the performance of the simple ORC configuration.

### 4.2. Parametric Study

In this section a parametric study was developed to visualize the performance of the system with the selected organic fluids and the performance of the parameters in the presence of vibration; this allowed us to determine the fluids that provided better results and had the best properties for the working conditions.

**Figure 4:**
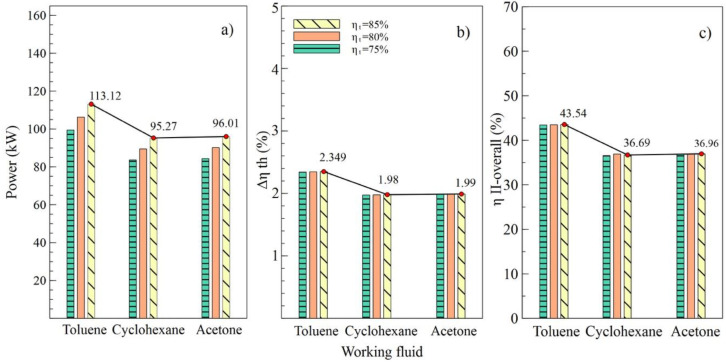
Influence of turbine efficiency on performance of simple ORC with different organic fluids; (**a**) Net power, (**b**) Absolute increase in thermal efficiency, (**c**) Overall exergetic efficiency.

**Figure 5:**
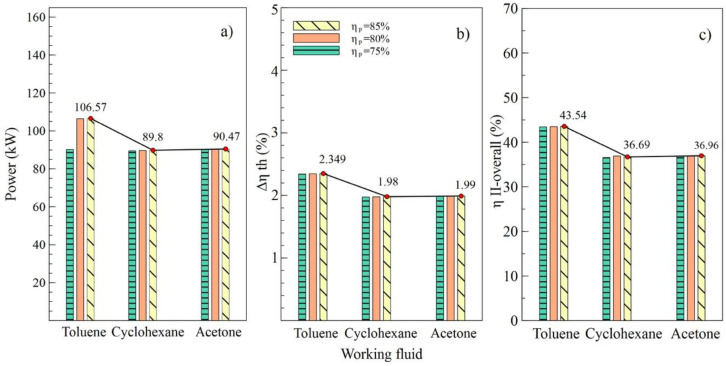
Influence of pump efficiency on performance of simple ORC with different organic fluids; (**a**) Net power, (**b**) Absolute increase in thermal efficiency, (**c**) Overall exergetic efficiency.

**Figure 6:**
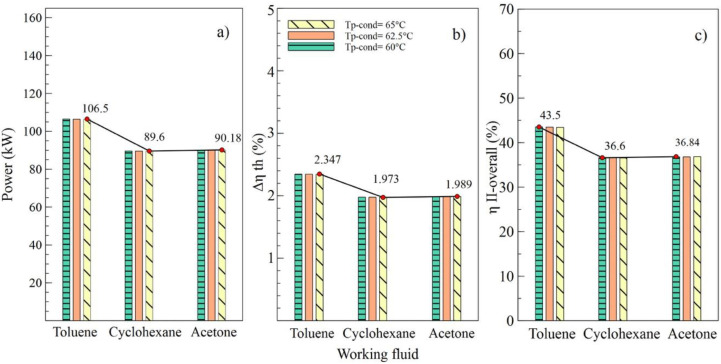
Influence of condenser pinch temperature on the performance of simple ORC with different organic fluids; (**a**) Net power, (**b**) Absolute increase in thermal efficiency, (**c**) Global exergetic efficiency.

**Figure 7:**
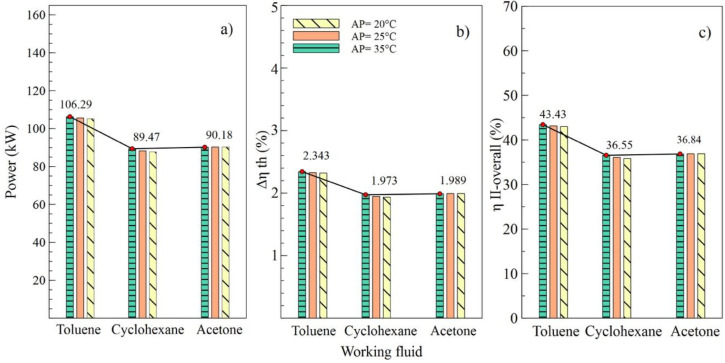
Influence of evaporator pinch temperature on the performance of simple ORC with different organic fluids; (**a**) Net power, (**b**) Absolute increase in thermal efficiency, (**c**) Overall exergetic efficiency.

Figure 4, Figure 5, Figure 6 and Figure 7 show the influence of variation on the performance parameters in the ORC cycle using toluene, cyclohexane, and acetone. The parameters selected to visualize the behavior of the ORC cycle were the net power, the absolute increase of the thermal efficiency, and the overall exergetic efficiency. Toluene was the fluid that guaranteed the best performance of the system in all parametric cases, as it maintained the highest values compared to other organic fluids.

Figure 4 shows the performance of the simple ORC cycle with the three fluids selected at different turbine efficiency values (T1). It can be noted that the maximum values of power, the absolute increase in thermal efficiency, and overall exergetic efficiency were obtained when the turbine efficiency was 85%. That is, as the efficiency of this equipment increased, increases in net power and all system efficiencies were observed. Figure 4a shows how the net power values changed concerning the variation of efficiencies for each fluid used. In the case of toluene, an increase from 75% to 85% in turbine efficiency allowed an 11.9% increase in the net power of the system, from 101.02 kW to 113.12 kW, specifically.

The values allow us to visualize the performance of the system corresponding to the variations in efficiency of the pump, as shown in Figure 5. It can be observed in Figure 4b,c that the values of $\Delta \eta_{\mathrm{th}}$ and $\eta_{\mathrm{II}.\mathrm{overall}}$, respectively, were similarly affected, indicating that the variation of efficiencies in the turbine and the pump had a similar influence on the thermal parameters of the system. On the contrary, net power was influenced by the variations, as shown in Figure 5a, which reached a maximum value of 106.57 kW with an 85% efficiency in the pump (B2).

Effects of the variations of the condenser pinch temperature are shown as in Figure 6. It can be observed that toluene was the fluid that guaranteed the best performance of the ORC cycle, supplying a better net power and higher efficiencies of the cycle. In addition, it is indicated that variations in condenser pitch values were not significant in the performance of the ORC. Figure 6a evinces that, with a condenser pinch temperature of 60 °C and toluene as the working fluid, the net power reached a maximum value of 106.5 kW, which is 18.86% higher than the value obtained using cyclohexane and 18.09% higher than that obtained with acetone. The influence of the working fluid and the variations of the evaporator pinch temperature on the performance of the ORC cycle can be observed as in Figure 7. The best results were obtained when AP increased to 35 °C, and toluene was used as the working fluid. It is asserted that the variations in temperatures pinch either in the condenser or the evaporator did not represent a determinant influence on the performance of the cycle because both variables maintained an inversely proportional relation. On the other hand, the working fluid did influence the performance of the ORC cycle, as is the case with net power.

The results obtained in the parametric study allow us to affirm that the organic fluid with the most suitable properties for the established working conditions is toluene. Therefore, for thermodynamic calculations, the values and properties corresponding to toluene as an organic fluid were taken for this research.

### 4.3. Results of The Optimization

The multiobjective optimization of the parallel plate heat exchanger was performed considering two objective functions with four decision variables, which are detailed as in Table 2. The selected variables correspond to the geometric parameters of the ITC2, all to obtain the optimal geometric configuration that guarantees the operational conditions and the thermodynamic requirements of the secondary thermal circuit.

**Figure 8:**
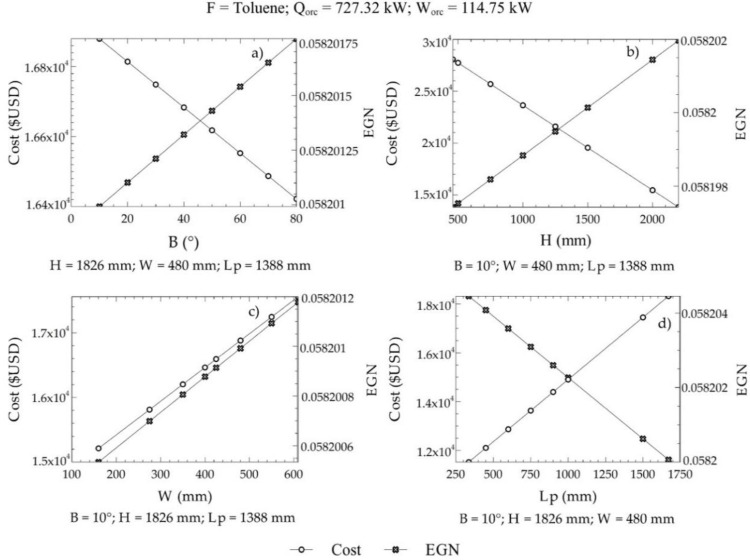
Variations of the EGN and the acquisition cost with the decision criteria; (**a)** Angle of inclination, (**b**) plate height, (**c**) Plate width, (**d**) Length between plates.

For this case, 130 commercial configurations were available to obtain the reference values of the objective functions. Positive exergy destruction in the three zones of the equipment was considered as a restriction ${\overset{\cdot }{e}}_{Z1}+{\overset{\cdot }{e}}_{Z2}+{\overset{\cdot }{e}}_{Z3}>0$. In addition, the angle of inclination of the plates ranged from 10° to 80°. On the other hand, the limits of C2, C3, and C4 were given by the geometric dispositions of the heat exchangers that can be purchased in the market with a determined cost. Figure 8 shows the variations of the EGN and the cost of acquisition with the decision criteria, where it can be observed that the angle of inclination, the height of the plate, width of the plate, and length between plates affected the selected objective functions (cost and EGN).

Figure 8 shows the influence of design parameters on both the cost of equipment acquisition and the amount of entropy generated within ITC2. An increasing angle of inclination in the grooves of the plates resulted in a lower cost of the equipment, while, at the same time, the entropy generated in the system increased, as in Figure 8a. This is caused by an increase in corrugation on the surface of the plate. Likewise, a similar behavior was shown with an increase in the height of the plate in the ITC2, as in Figure 8b, for variations in the cost of the equipment and variations in the amount of entropy generated. Increasing the width of the ITC2 plate created oversizing, which generated high manufacturing costs and, therefore, acquisition of equipment, causing a significant increase in its values. Similar behavior occurred in the generation of entropy, almost with the same inclination as in Figure 8.

On the other hand, an increasing length between the plates decreased the number of plates required for the heat transfer area. Therefore, an increase in this parameter represents a decrease in the pressure drop per flow, which minimizes the amount of entropy generated during the process (Figure 8d). In our case, it represents a critical design parameter because the acquisition cost increases due to an oversizing of the equipment, which represents a problem for the optimization of this objective.

**Figure 9:**
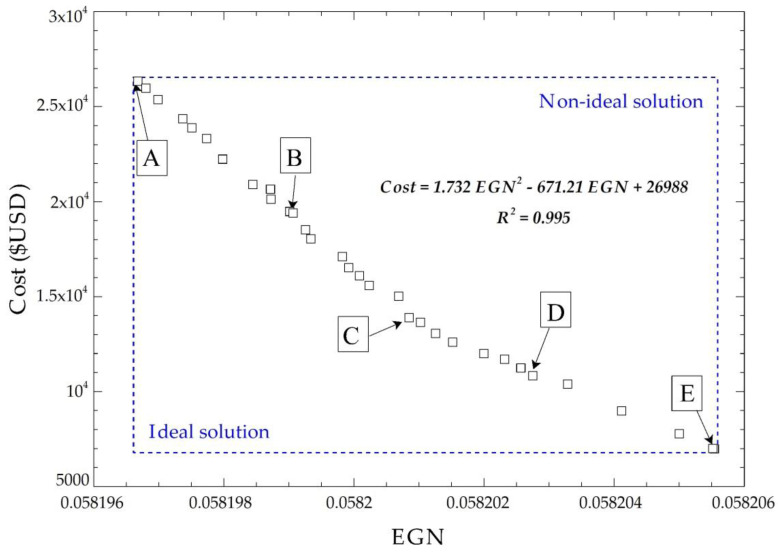
Pareto frontier for the cost of acquisition with EGN of the system.

The multiobjective optimization solution does not have a global optimal point because no single response exists that simultaneously optimizes each objective variable. Therefore, a procedure of selecting the final optimal values by applying the NSGA II [41,42] method is included, where 32 points are obtained from the objective functions after performing 230 possible iterations. Figure 9 shows the Pareto frontier for the cost of acquisition with the exchanger EGN, plus five possible solution points.

The optimal points have a linear tendency; this is due to the order of the related objective functions in the optimization process, which are of the first order because of the correlation that exists between the geometric parameters.

The five points selected at the Pareto frontier meet the optimization requirements, as shown in Figure 9. Applying TOPSIS gives the ideal positive and negative solutions to find the optimal points (Table 3).

**Figure 10:**
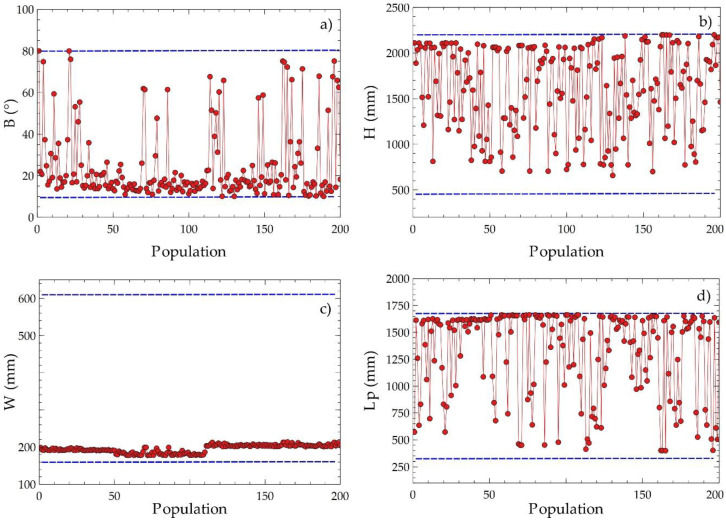
Disperse distribution of decision criteria for EGN and acquisition cost with population on Pareto frontier; (**a**) C1, (**b**) C2, (**c**) C3, (**d**) C4.

Figure 10 presents the dispersed distributions of the design variables to obtain information on the evolution of their values during the optimization process. The angle of inclination had a disperse distribution with a tendency close to the lower limit of the domain, as shown in Figure 10a, which suggests that the variable plays a significant role in the compensation of the objective variables. The plate width value tended to approximate the lower values around 200 mm, as shown in Figure 10c, indicating that decreasing this parameter improves the optimization result. The plate height and the length between plates had scattered distributions with tendencies to a higher value, as shown in Figure 10b,d. The observations obtained suggest that these design variables are determinant for identifying the critical points of the objective functions proposed for equipment optimization.

The multicriteria decision technique indicated that point D is nearest to the ideal solution. From this point, the optimal values of the decision criteria are obtained and are shown in Table 4.

The optimal values of the geometric parameters of ITC2 form a transfer area of 22.04 m^2^, which ensures a heat transfer flow from the thermal oil to the toluene of 693.87 kW. An overall thermal engine efficiency of 41.6% was achieved for the ORC cycle as a result of the minimum amount of possible exergy destroyed by both temperature and pressure drops.

## 5. Conclusions

This work presents a model to optimize a parallel plate heat exchanger (ITC2), which corresponds to the evaporator of a secondary thermal circuit, through the NSGA-II approach. The acquisition cost of the equipment and the entropy generated were selected as objective functions, whereas the geometric parameters of the exchanger were considered as decision variables. As a single parameter value cannot satisfy both functions to be optimized, a series of optimal points are presented in the form of the Pareto frontier, which represents the equilibrium curve between both functions. The working fluid has a determinant role in the performance of the simple ORC cycle, so it is necessary to select the organic fluid that provides more significant benefits to the system. A study on performance parameters of the system is carried out before varying the efficiency of both the turbine and the pump as well as the temperature pinches of both the evaporator and the condenser of the system. With this, we attempted to identify which organic fluid had the highest net power values, the highest absolute increase in the thermal efficiency, and highest overall exergetic efficiency. Effects of the decision criteria on the objective functions are also studied by means of a sensitivity analysis, which showed that the length between plates is the most promising criteria, since its increase causes an elevation in the costs of the equipment up to a maximum value of USD 18,000 and a decrease in the entropy generation number.

The results found that, when applying the methodology proposed for this evaporator design through multiobjective optimization and selecting the best configuration of the five possible solutions through the TOPSIS method, point D was the best solution according to the established criteria. It was possible to minimize the entropy generation number (NGE = 0.058) and the acquisition cost of the equipment (USD 10,385.55), with an inclination angle (20.44°), plate height (2070.32 mm), plate width (205.16 mm), length between plates (800.49 mm), and heat transfer area of 22.04 m^2^. This guarantees that heat transferred from the thermal oil (Therminol 75) to the toluene is 693.87 kW, overall thermal motor efficiency of the ORC cycle is 41.6%, and pressure drop is 980.32 mbar, which is within the permissible backpressure range of the engine.

The results of optimization may vary with the change of some configurations in the genetic algorithm, for instance: the fraction of the population, the population number, and the number of allowed iterations. In this case, optimization was performed with a total of 200 particles in the iterative space fractionated, achieving a value of 0.16 for a total of 32 optimal points, as shown in the Pareto frontier. In conclusion, the optimal geometry will be different for each case because there are infinite combinations in the iteration space, and adjustments were made in the configuration of the optimization model.

This proposed methodology can be applied to the thermodynamic and economic optimization of plate heat exchangers in any type of heat recovery system with indirect evaporation of the organic fluid. This methodology is always more relevant for cases where there are limitations in heat source backpressure, such as industrial engines with medium and high exhaust gases temperatures, and is applicable in cases where ORC technology has not been widely applied commercially.

## Figures and Tables

**Table 1:** Parameters of an integrated recovery system with motor and single ORC for selected fluids.

Fluid	$\overset{\cdot }{W}net$	$\eta_{\mathrm{th}}\;\mathrm{Engine}/\mathrm{ORC}$	$\Delta \eta_{\mathrm{th}}$	$\eta_{\mathrm{th}}\;\mathrm{ORC}$	$\varepsilon_{\mathrm{hr}}$	$\eta_{\mathrm{II}-\mathrm{overal}}$	BSFC	$\overset{\cdot }{e}\;\mathrm{Total}$
	(kW)	(%)	(%)	(%)	(%)	(%)	(g/kWh)	(kW)
Acetone	100.47	40.80	2.21	19.51	7.96	41.05	169.63	114.97
C_6_H_12_	84.94	40.46	1.87	16.50	6.73	34.71	169.50	127.95
Toluene	84.46	40.45	1.86	16.40	6.69	34.51	169.50	128.90
n-heptane	71.72	40.17	1.58	13.92	5.68	29.30	169.89	139.59
n-octane	65.33	40.03	1.44	12.69	5.17	26.69	169.24	145.43
n-nonane	59.74	39.91	1.32	11.60	4.73	24.41	169.29	150.59
n-decane	54.86	39.80	1.21	10.65	4.35	22.41	169.54	155.13
MDM	48.90	39.67	1.08	9.50	3.87	19.98	169.67	159.36
D4	45.83	39.61	1.02	8.90	3.63	18.72	169.77	162.61
MD2M	41.24	39.50	0.91	8.01	3.27	16.85	169.87	166.35
D5	39.02	39.46	0.87	7.58	3.09	15.94	169.89	168.38
MD3M	33.97	39.35	0.76	6.60	2.69	13.88	169.93	172.72
D6	32.04	39.30	0.71	6.22	2.54	13.09	169.95	174.16
MD4M	30.84	39.28	0.69	5.99	2.44	12.60	169.96	175.82

**Table 2:** Variables decision on multi-objective optimization.

Variable Decision	Symbol	Unit	Minimum Value	Maximum Value	Criteria
Angle of inclination	β	°	10	80	C1
Plate height	H	mm	460	2200	C2
Plate width	W	mm	160	610	C3
Plate spacing	Lp	mm	336	1671	C4

**Table 3:** Optimized values of parameters and objective functions.

Parameter of Design	Unit	Base State	Optimal Values				
			A (min EGN)	B	C	D	E (min cost)
β	°	10	10.05	18.23	14.32	20.44	74.60
H	mm	1826	658.29	1462.94	2112.66	2070.32	2198.65
W	mm	480	201.09	203.87	204.79	205.16	212.52
Lp	mm	1388	1652.38	1593.48	1500.74	800.49	400.14
$A_{plate}$	m^2^	0.876	0.132	0.298	0.4325	0.424	0.467
$N_{Plate}$	-	35	360	90	52	52	48
$A_{t}$	m^2^	30.6	47.52	26.82	22.49	22.04	22.42
$\overset{\cdot }{Q}$	kW	693.87	693.87	693.87	693.87	693.87	693.87
${\overset{\cdot }{W}}_{net}$	kW	114.75	114.75	114.75	114.75	114.75	114.75
$\eta_{\mathrm{ORC}}$	-	0.41	0.41	0.41	0.41	0.41	0.41
$V_{\mathrm{AT}}$	m/s	0.146	0.0423	0.156	0.253	0.253	0.262
$V_{\mathrm{ORC}}$	m/s	1.509	0.437	1.613	2.615	2.612	2.705
$Re_{\mathrm{AT}}$	-	1029.03	245.62	969.11	1669.78	1668.07	1747.41
ReORC	-	12,017.13	2,868.33	11,317.45	19,499.90	19,479.93	20,406.45
$h_{4ORC}$	kjkg	1596.7	946.47	1758.86	2172.31	2171.12	2196.62
$U_{Z2\_C}$	Wm2K	996.95	696.24	1141.11	1309.68	1309.99	1313
$jf_{Z2\_AT}$	-	0.0748	0.115	0.0762	0.0647	0.0647	0.0638
$jf_{Z2\_ORC}$	-	4.698	17.05	4.823	2.985	2.95	4.38
$\Delta PF_{Z2\_AT}$	mbar	22.13	4.12	33.55	66.37	35.34	18.45
$\Delta PF_{Z2\_ORC}$	mbar	845.8	371.92	129.28	1863.6	980.32	772.5
$Vin_{Z1\_AT}$	m/s	4.024	4.024	4.024	4.024	4.024	4.024
$Vin_{Z1\_ORC}$	m/s	1527.42	1527.42	1527.42	1527.42	1527.42	1527.42
Cost	$ USD	16,954.07	26,335.14	19,406.80	13,644.11	10,385.55	6,979.19
EGN	-	0.058219	0.058197	0.058199	0.058201	0.058203	0.058206

**Table 4:** Multi-objective optimal solution.

Objective Function	Optimal Value	Value of The Decision Variables			
		C1	C2	C3	C4
Equipment cost ($ USD)	10,385.55	20.44	2070.32	205.16	800.49
EGN	0.05820328				

## Abbreviations

The following abbreviations are used in this manuscript:

BSFC	Brake-Specific Fuel Consumption
EGN	Entropy Generation Number
ICE	Internal Combustion Engine
LMTD	Logarithmic Media Temperature Difference
ORC	Organic Rankine Cycle
WHR	Waste heat recovery
**Nomenclature**	
A	Area (m^2^)
$A_{\mathrm{plate}}$	Plate area (m^2^)
$A_{Z1}$	Heat transfer area zone 1 (m^2^)
Cp	Specific heat at constant pressure (J/kg K)
D	Diameter (m)
$\overset{\cdot }{e}$	Destroyed exergy (kW)
E	Energy (J)
ex	Specific exergy (kJ/kg)
$ex_{G}^{\mathrm{ch}}$	Chemical exergy of exhaust gases (kJ/kg)
H	Plate height (mm)
h	Specific enthalpy (kJ/kg)
jf	Heat transfer Colburn factor
K	Thermal conductivity (W/m·K)
L	Length (mm)
LHV	Lower heating value (kJ/kg)
Lp	Length between plates (mm)
m	Mass (kg)
$\overset{\cdot }{m}$	Mass flow (kg/s)
$N_{\mathrm{Plate}}$	Number of plates
Nu	Nusselt number
Pinch	Minimum temperature difference (°C)
Pr	Prandtl number
$\overset{\cdot }{Q}$	Heat transfer flow (kW)
R	Universal gas constant (atm L/mol K)
Re	Reynolds number
S	Entropy (kJ/K)
$\overset{\cdot }{S}\mathrm{gen}$	Generated entropy (kW)
T	Temperature (K)
U	Overall heat transfer coefficient (W/m^2^K)
v	Velocity (m/s)
W	Plate width (mm)
${\overset{\cdot }{W}}_{\mathrm{net}}$	ORC Power (mbar)
$\Delta P_{t}$	Pressure drop on the tube side (mbar)
$\Delta P_{c}$	Pressure drop on the shell side (mbar)
ΔPin	Inlet pressure drop (mbar)
ΔPF	Flow pressure drop (mbar)
$X_{i}$	Molar gas fraction
**Greek Symbol**	
β	Angle of inclination
ρ	Density (kg/m^3^)
$\varepsilon_{\mathrm{hr}}$	Heat recovery efficiency
$\eta_{I.c}$	Thermal efficiency of the cycle
$\Delta \eta_{\mathrm{th}}$	Absolute increase in thermal efficiency
$\eta_{I.\mathrm{overall}}$	Overall energy conversion efficiency
$\eta_{\mathrm{II}.\mathrm{overall}}$	Overall exergetic efficiency
$\eta_{\mathrm{II}.\mathrm{ORC}}$	Exergetic efficiency based on the second law of thermodynamics
υ	Kinematic viscosity (m^2^/s)
**Subscript**	
0	Reference
AT	Thermal oil
EXT	External
G	Exhaust gases
TOT	Total
Z1	Preheating zone in ITC2
Z2	Evaporation zone in ITC2
Z3	Overheating zone in ITC2
